# Lion in Sheep’s Clothing: Glioblastoma Mimicking Intracranial Hemorrhage

**DOI:** 10.7759/cureus.14212

**Published:** 2021-03-31

**Authors:** Andrea Broka, Zhenisa Hysenaj, Shorabh Sharma, Razia Rehmani

**Affiliations:** 1 Internal Medicine, St Barnabas Hospital Health System, Bronx, USA; 2 Radiology, St Barnabas Hospital Health System, Bronx, USA

**Keywords:** intracranial hemorrhage, glioblastoma, cancer, seizure, syncope

## Abstract

Intracranial hemorrhage (ICH) as a unique image finding, is a rare presentation of glioblastoma (GBM), and can pose a diagnostic challenge. Hypertensive vascular changes are responsible for the majority of the ICH cases, where hemorrhage from brain tumors account only for 5.1% to 7.2% of cases and, the etiology seems to be multifactorial. We present a clinical case of a 70-year-old male who came to the emergency department after a syncopal episode at the workplace, associated with nausea and vomiting. Computed tomography scan and magnetic resonance imaging showed intracranial subdural hematoma, subarachnoidal and interventricular hemorrhage without any underlying lesion. Follow-up imaging in one month showed a new ICH with a thick peripheral mass concerning an underlying neoplasm. The patient underwent tumor resection and immunohistochemical staining confirmed glioblastoma. Despite a multiapproach treatment, including, chemotherapy, radiotherapy, and follow-up surgery, the outcome was poor. GBM is a great mimicker and may initially present with unassuming intracranial hemorrhage with a much more sinister hidden diagnosis. A high index of suspicion on initial imaging based on the patient’s demographics with early tissue diagnosis is crucial in arriving at the correct diagnosis. This case reinforces the importance of close interval follow-up in patients with spontaneous ICH, maintaining a high suspicion for brain tumors. To date, GBM remains a poor prognosis despite combined surgery, chemotherapy, and radiotherapy treatment.

## Introduction

Glioblastoma (GBM) (previously known as glioblastoma multiforme) is a primary brain tumor derived from the neoplastic transformation of glial cells. As per World Health Organization (WHO) classification, GBM is a grade IV glioma and accounts for more than 10,000 cases a year with a poor survival rate, up to 15 months. They usually occur after the age of 40 with a peak at 65 to 75 years and present with gradual, worsening focal neurological deficits, and/or seizure [1]. In rare cases, abrupt neurologic compromise due to intracranial bleeding can be the only finding on imaging (0.54%-3.4% of the cases [2]) and can pose a diagnostic challenge.

## Case presentation

**Figure 1:**
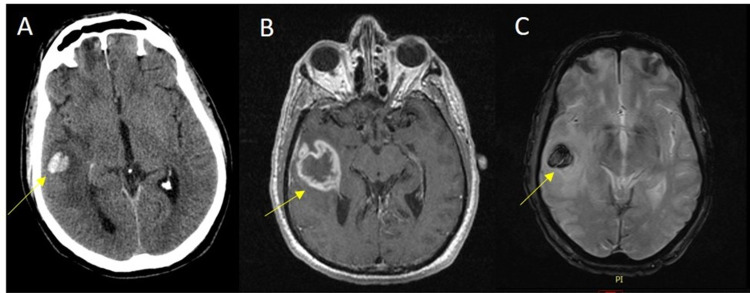
Axial noncontrast CT (A) demonstrates intraparenchymal presumed hematoma in this post-traumatic patient. Axial postcontrast T1 MRI image (B) shows a thick rim enhancing focus at the site of hemorrhage, which is confirmed by blooming on GRE series (C). There is surrounding edema. GRE: gradient recall echo.

We present a case of a 70-year-old male with no pertinent past medical history who presented to the ED after a syncopal episode at the workplace, preceded by headaches and vision changes. The patient was brought to the emergency room where he was found to be in hypertensive emergency, complaining of lightheadedness and episodes of vomiting. The physical exam was unremarkable. Initial CT head showed intracranial subdural hematoma, subarachnoidal and interventricular hemorrhage. Further trauma imaging was negative. Further imaging computed tomography angiography (CTA) head and neck showed stable intracranial hemorrhage, without evidence of aneurysm, high-grade stenosis, or occlusion. Magnetic resonance (MR) brain on initial admission showed multiple subacute resolving hemorrhages. A focal 1.5 cm rim-enhancing lesion about the right posterolateral temporal lobe with associated blooming on gradient recall echo (GRE) and T2 prolongation was presumed to be a subacute hemorrhagic contusion in the setting of trauma (Figure 1A-C).

A month later, while undergoing follow-up CT head, a new temporal acute hemorrhage was noted and the patient was hospitalized to the medical intensive care unit. His main complaint was intermittent weakness of the left upper extremity since discharge, but the physical and neurological examinations were unremarkable.

Due to the unclear etiology of ICH, the patient had a repeat MRI. Much to our surprise, this follow-up MRI showed a significant increase in the size of the previously seen right temporal hematoma with heterogeneous thick peripheral mass-like enhancement with areas of hemorrhage which extended to the adjacent dura. This much uglier appearance and rapidly increasing size were now concerning for either recurrent bleed perhaps into an underlying primary or metastatic hemorrhagic neoplasm.

The patient underwent right temporal craniotomy with tumor resection. Pathology showed glioblastoma, WHO grade IV, negative for IDH1 R132H mutation by immunohistochemistry, no epidermal growth factor receptor (EGFR) amplification. Additional molecular workup: positive for Telomerase reverse transcriptase promoter mutation, negative for expression of EGFR deletion mutant. No MGMT methylation detected.

The postoperative course was uncomplicated, and the patient was discharged home to follow up with Medical and Radiation Oncology. He completed adjuvant chemoradiation therapy with oral temodar and was on monthly pulsed adjuvant temozolomide (TMZ). 

**Figure 2:**
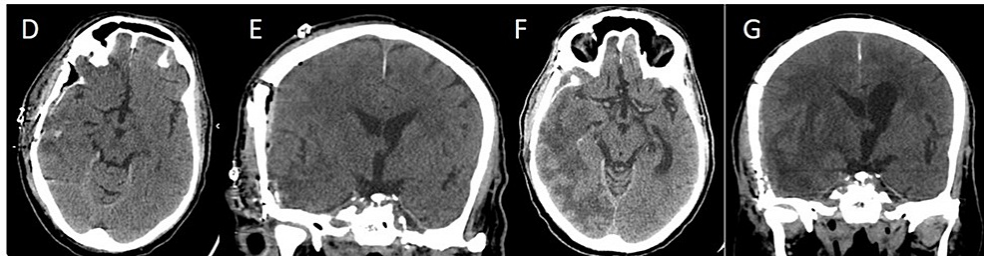
Axial (D) and coronal (E) noncontrast CT images demonstrate post-surgical changes after right-sided craniotomy with evaluation of the hematoma. However, follow-up CT (axial (F) & coronal (G)) nine months later, demonstrate marked right cerebral hemisphere edema centered at right temporal lobe with mass effect on the right lateral ventricle with right to left midline shift.

**Figure 3:**
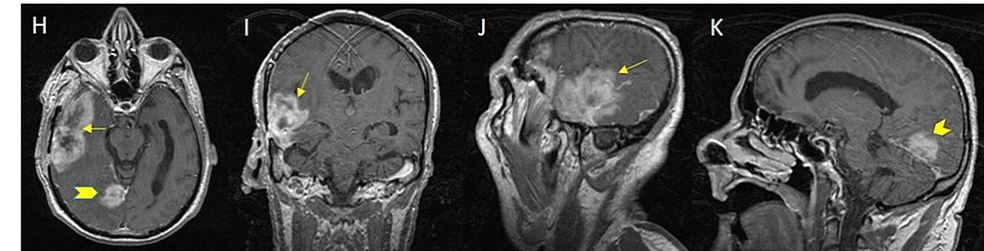
Axial (H), coronal (I) and sagittal (J, K) T1 images from post contrast MRI demonstrate an aggressive appearing markedly heterogenous large mass in the right middle cranial fossa involving the right temporal lobe (arrow). An additional separate lesion is seen adherent to the right side of the tentorium (arrowhead). This was found to be a recurrent glioblastoma multiforme mimicking as hemorrhage before.

Nine months after tumor resection and whilst still on therapy, the patient was admitted for a new-onset seizure. Imaging revealed tumor recurrence at the surgical bed in the temporal lobe with an additional lesion in the right occipital lobe (Figures 2, 3D-K). The patient underwent surgical resection for the second time. Thereafter, he was shortly readmitted with lethargy, fatigue, and confusion. At this point, the family opted for palliative care, and a home hospice program was set up and no further treatment was pursued. 

## Discussion

We present a teaching case report of a challenging GBM diagnosis that mimicked intracranial hemorrhage at onset in the setting of new-onset syncope and fall. GBM represents the most extreme tumoral transformation of the glial cell. Overt primary ICH is an infrequent presentation and is associated with abrupt onset of symptoms. Intracranial hemorrhage as part of the hemorrhagic stroke represents the second most common subtype of stroke seen in the emergency setting and accounts for 10% to 20% of cases [3]. Hypertensive vascular changes are responsible for the majority of the cases, where hemorrhage from brain tumors account only for 5.1% to 7.2% of cases [2,4].

Common radiologic findings are poorly defined intra-axial lesions with mass effect, involving white matter tracts or large tumors with thick, irregular-enhancing rings and a central necrotic core, sometimes with hemorrhagic changes and perilesional vasogenic edema. The term Multiforme was coined to describe the above presentation in 1926 by Percival Bailey and Harvey Cushing [5]. 

A retrospective study done by Navi et al. in brain cancer patients showed that intracranial hemorrhage manifest by multifocal intracerebral compartment bleeding (87% of cases) was the most common presentation. The mechanism of bleeding can be related to the direct effect of neoplastic cells affecting blood vessels, or malformed vasculature proliferation due to hypoxia, tumor necrosis, but can also be related to tumor coagulopathy. Instead, hypertension is considered to be responsible for only 8% of parenchymal hemorrhages in cancer patients [6].

According to the literature review for malignancy as a cause for spontaneous intracerebral hemorrhage done by Joseph et al. of 2017, out of 1,445 patients diagnosed with ICH from 1978 to 2010, only 33 patients (2.2%) presented with ICH as the initial findings of an undiagnosed tumor [7,8]. GBM presenting as ICH accounted for 18 cases and a diagnostic delay of a median of 60 days from the presentation was noted. The confirmatory diagnosis was done either by pathology or other imaging modalities. Imaging techniques used for diagnosis were variable and not comparable due to new advanced technology available in recent years.

In a retrospective study done by Thaler et al. normal neuroimaging was found in 8 of 102 patients with GBM. These patients presented with seizures, weakness, or cognitive disturbances. Most of these patients underwent repeat neuroimaging within six months, which then showed progressive disease with confirmatory biopsy [8].

The diagnostic imaging of choice for an intracranial neoplastic disease is MRI with and without contrast. Multitudes of advanced imaging sequences such as functional MRI, diffusion-weighted imaging, diffusion tensor imaging, dynamic contrast-enhanced MRI, perfusion imaging, proton magnetic resonance spectroscopy as well as positron-emission tomography play a key role in the diagnostic process. In a recent meta-analysis published in 2017 by van Dijken, sensitivity and specificity can vary from 68% and 77%, respectively, on anatomical MRI to 91% and 95% on magnetic resonance spectroscopy [9]. 

Surgery is the initial therapeutic approach for GBM and remains a hallmark in the treatment of malignant brain tumors. GBM remains a poor prognosis despite surgical resection and combined chemotherapy and radiotherapy. To date, excluding Tumor-Treating Fields (TTFields), no new agents improve survival when added to standard therapy. MGMT promoter methylation is predictive of response to Temozolomide, its role in the choice of first-line therapy is currently limited to the elderly GMB patients. No standard of care is established in the recurrent setting. Bevacizumab impacts progression-free survival, but its role in overall survival remains less certain. TMZ re-challenge is a treatment option, especially for O6-methylguanine-DNA methyltransferase promoter methylated GBM [10].

When tumor recurs, treatment options include supportive care, reoperation, re-irradiation, systemic therapies, and combined modality therapy [11]. In this setting, the role of reoperation remains unclear. A recent review of the literature, including 28 studies and 2,279 patients, who underwent a second surgery, showed a median survival from reoperation of 9.7 months and concluded that the extent of resection at reoperation improves overall survival, even in patients with subtotal resection at initial surgery [12].

In the case of our patient GMB re-occurred nine months after the first resection and the second surgery did not improve overall survival, patient was ultimately enrolled in hospice care and passed away shortly thereafter.

## Conclusions

GBM is a great mimicker. It may initially present with unassuming intracranial hemorrhage with a much more sinister hidden diagnosis. A high index of suspicion on initial imaging based on the patient’s demographics with early tissue diagnosis is crucial in arriving at the correct diagnosis. Close interval follow-up after surgery is a must since it has a very high rate of recurrence. MRI with and without contrast is the basic workhorse, however advanced MRI imaging such as MRI spectroscopy can be particularly helpful in early diagnosis. 
